# The unusual location of primary hydatid cyst: A case series study

**DOI:** 10.1515/med-2024-1030

**Published:** 2024-10-18

**Authors:** Seerwan Hama Shareef Qaradaghy, Diyaree Nihad Ismael, Shorsh Hama Hussein Ameen, Kawa Abdula Mahmood, Ismael Hama Amin Aghaways, Fadhluddin Nasruddin Shakor, Zana Othman Abdullah, Kawa M. Ibrahim, Mohammed Amin Ali Omer, Sangar Abdullah Mohammed, Aram Ahmed Mohammed, Safeen Hama Rasheed

**Affiliations:** Department of Surgery, College of Medicine, University of Sulaimani, Sulaimaniyah, Iraq; Department of Surgery, Sulaimani Teaching Hospital, Sulaimani Directorate of Health, Sulaimaniyah, Iraq; Department of Surgery, Shorsh Teaching Hospital, Sulaimani Directorate of Health, Sulaimaniyah, Iraq

**Keywords:** hydatid cyst, case series study, cystectomy, unusual location, hospital data

## Abstract

**Background:**

Cystic echinococcosis mainly affects the liver and lungs, in which the larvae from the microvascular wall in the liver pass to the lungs and then to the blood circulation and settle in any tissue or organ.

**Objectives:**

The objective of this study was to report the unusual location of hydatid cysts in infected patients in Sulaimaniyah City, Iraq.

**Patients and methods:**

This retrospective case series study enrolled 13 patients. They underwent a surgical operation to excise their cyst after confirmed diagnosis with blood investigations, electrocardiogram, chest X-ray, computed tomography scan, and magnetic resonance imaging (when needed). After the operation, the cyst was confirmed with histopathological examination, and patients were advised to take an Albendazole tablet.

**Results:**

Most patients were females from rural areas, with a mean age of 38.93 ± 14.4 years. Patients presented with cysts on the skin of the anterior abdominal wall, gluteal region, mesenteric area, pericardium, tibia bone meta diaphysis, right inguinal region, right thigh, skin of the anterior neck, spleen, left suprarenal gland, right breast, and the iliopsoas muscle.

**Conclusions:**

The hydatid cyst can affect any body part with no site immune and often produces nonspecific symptoms.

## 1 Introduction

Hydatidosis is the most common parasitic disease in humans and animals, and humans are the accidental hosts. This disease has a global spread, especially in endemic and hyper-endemic areas and the Mediterranean region. It has excellent medical, veterinary and economic importance [1,2].

Hydatid cyst diseases among humans are caused by infection at the metacestode stage of *Echinococcus*, especially *E. granulosus* and *E. alveolaris*. Canine, especially dogs and wolves, are primary carriers, while sheep, cattle, and deer are the intermediate hosts. Humans are accidental hosts that do not play a role in the biological cycle and are infected by ingesting ova from soil/water contaminated by faeces [3,4]. Cystic echinococcosis may disturb all organs, but mostly the liver/lungs, and the larvae can pass from these organs to the systemic circulation and reach tissues in 10–20% of cases, [5] but the mechanism is still controversial [6]. The larvae can also diffuse to the venous mesenteric lymph vessels and settle in various intra-abdominal organs by transmural migration from the intestinal wall [7].

Although hydatid cysts usually have no clinical symptoms in the early stages of infection in humans, clinical signs appear gradually due to the growth of cysts and mechanical pressure. The severity of the disease and its manifestations depend on the infected organ, the infection’s severity, and the infected organ’s sensitivity to the cyst. The pressure of single-cavity cysts on the vessels sometimes causes the vessel to rupture, bleed, and cause mechanical injury. The larger the cyst, the more pressure it puts on the surrounding tissues, causing atrophy or disrupting their normal functions [8,9]. Finding hydatid disease with atypical localization and the liver/lungs (secondary atypical) is easy to comprehend. Atypical localization without hydatid disease in the liver and lung (primary atypical) is a separate subject of discussion. It is difficult to explain why larvae may directly pass through the intestinal system to the systemic circulation without going to the liver or forming a cyst [10].

Cystic echinococcosis is common in areas where agriculture and raising animals are frequent, and hydatid disease continues to be a severe public health problem in many developing countries. In Iraq, the incidence is about 6.3 in 100,000 population [11]. Recently, the World Health Organization (WHO) conducted hydatidosis in the subgroup of neglected diseases and has put control programs in place [12]. Liver and lung hydatid cysts represent about 90% of all hydatid cyst diseases; the other organs are affected less frequently, and the exact incidence is difficult to estimate. Unusual primary hydatid disease can occur via hematogenous or lymphatic spread [13]. An unusual location of hydatid disease has been studied worldwide, such as in Tunisia (retrovesical hydatid cysts) [14], Turkey (kidney, heart, bone, bladder, pancreas, and spleen) [15], and India (kidney, bone, brain, spleen, muscle, skin, peritoneal, and pelvic cavity) [16].

Like other Iraq cities, Sulaimaniyah City is endemic to hydatid cysts [11]. Thus, in this retrospective study, we reported the location of primary atypical hydatid cysts in patients in Sulaimaniyah City.

## 2 Patients and methods

### 2.1 Study design and setting

This retrospective case series study was conducted from 1994 to 2018 at Sulaimani Surgical Teaching Hospital based on 13 patients’ case sheet records (consecutive cases).

### 2.2 Inclusion criteria

Patients with unusual locations of hydatid cysts, regardless of age, gender, and nationality, were included in the study.

### 2.3 Exclusion criteria

Patients presented with liver and lung hydatid cysts (secondary atypical hydatid cysts) were excluded.

### 2.4 Pre-intervention consideration

Initially, patients were checked for vital signs. Then, blood investigations, electrocardiogram (ECG), chest X-ray, computed tomography (CT) scan, and magnetic resonance imaging (MRI) were done for patients to confirm the diagnosis. Consequently, they underwent a surgical operation to remove the cyst under general (*n* = 9) and spinal (*n* = 4) anaesthesia. The type of incision depended on the anatomical locations of the cyst, with cosmetic results being considered.

### 2.5 Types of intervention(s) employed

Nine patients underwent operation under general anaesthesia and four under spinal anaesthesia, in which 11 were supine, and 2 were prone. As a result of the suspicion of a hydatid cyst, the recommended precautions were followed in the operation theatre. Therefore, they were nil by mouth for 6 h before the intervention.

### 2.6 Peri-intervention consideration

During surgical operations, the patients were followed up through ECG monitoring. The intravenous fluid in the form of crystalloids was given according to clinical judgment by the anaesthetist. The required precautions were taken intraoperatively using Yankauers suckers and sterilization to prevent spillage of daughter cysts. The operation was performed by a team of specialist surgeons, senior house officers, and nurses.

### 2.7 Post-intervention consideration

After the operation and confirmation of diagnosis by histopathological examination, all patients were advised to use Albendazole tablets to prevent the parasites from growing. The recommended dose for individuals >60 kg was 400 mg/orally/twice daily for 28 days and then 14 drug-free days for 3 cycles. Patients <60 kg were advised to use 15 mg/kg orally/daily for 28 days (not more than 800 mg/day) and then 14 drug-free days for three cycles.


**Ethics approval and consent to participate:** Ethics approval was obtained from the Medical Ethics Commission for Clinical Studies in the Sulaimani Teaching Hospital. All procedures were followed according to Helsinki’s declaration.
**Informed consent:** Written informed consent for publication was obtained from the patients before publication at admission.

## 3 Results

### 3.1 Participants

This case series included 13 patients aged 16–70 years who had a mean age of 38.93 ± 14.4 years with a median age of 35.15 years, and most of them were from a rural area (Table 1). The most common symptom among patients was pain with feeling mass (only two patients).

**Table 1:** Demonstrating age, gender, and anatomical location of primary atypical hydatid cyst in studied cases

Case number	Age (years)	Gender	Cystic location
1	35	Female	Mesentery
2	24	Female	Right inguinal
3	21	Female	Pancreas
4	55	Female	Subcutaneous tissue of the anterior abdominal wall
5	45	Male	Left tibia
6	39	Female	Thigh
7	31	Female	Neck
8	60	Female	Left suprarenal gland
9	35	Female	Spleen
10	33	Female	Right breast
11	38	Male	Left gluteal
12	16	Female	Mesentery
13	25	Male	Scalp

### 3.2 Patient number 1 with mesenteric hydatid cyst

**Figure 1:**
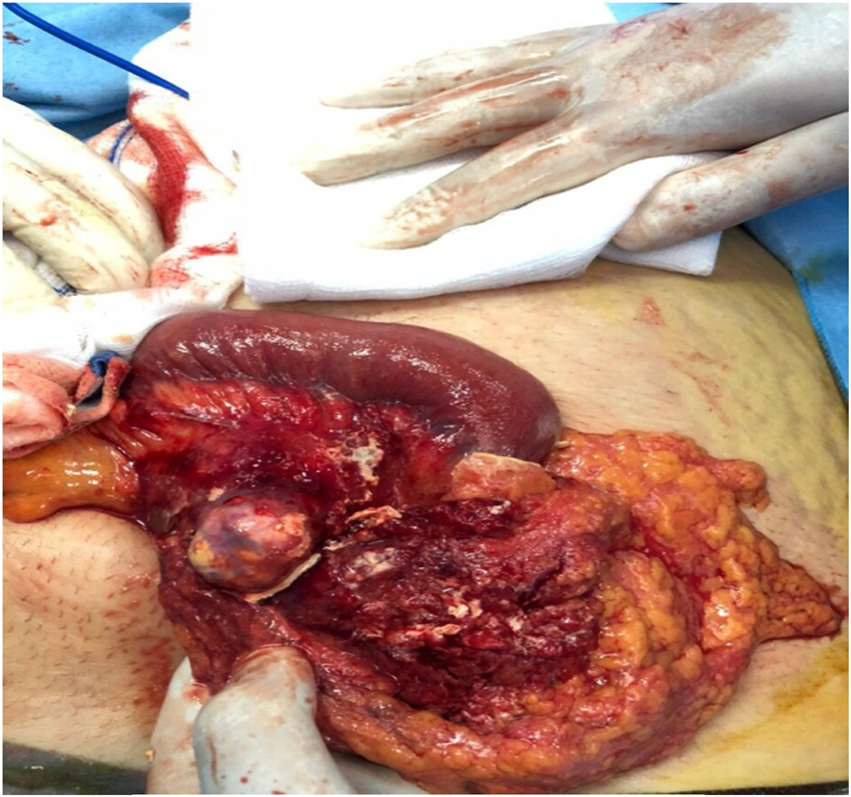
A mesenteric hydatid cyst (end cystectomy) was reported in patient number 1.

A 35-year-old female presented with mild nonspecific abdominal pain for 6 months, associated with nausea but no vomiting, no history of weight loss, and no constipation. General and abdominal examinations were unremarkable, with normal laboratory investigations. Ultrasound (U/S) showed an intra-abdominal cystic lesion, and a CT scan showed a hydatid cyst of the mesentery. By midline, laparotomy abdomen was opened, all intra-abdominal organs were checked, and there was only a cystic lesion in the mesentery (small bowel) (Figure 1).

### 3.3 Patient number 2 with right inguinal hydatid cyst

**Figure 2:**
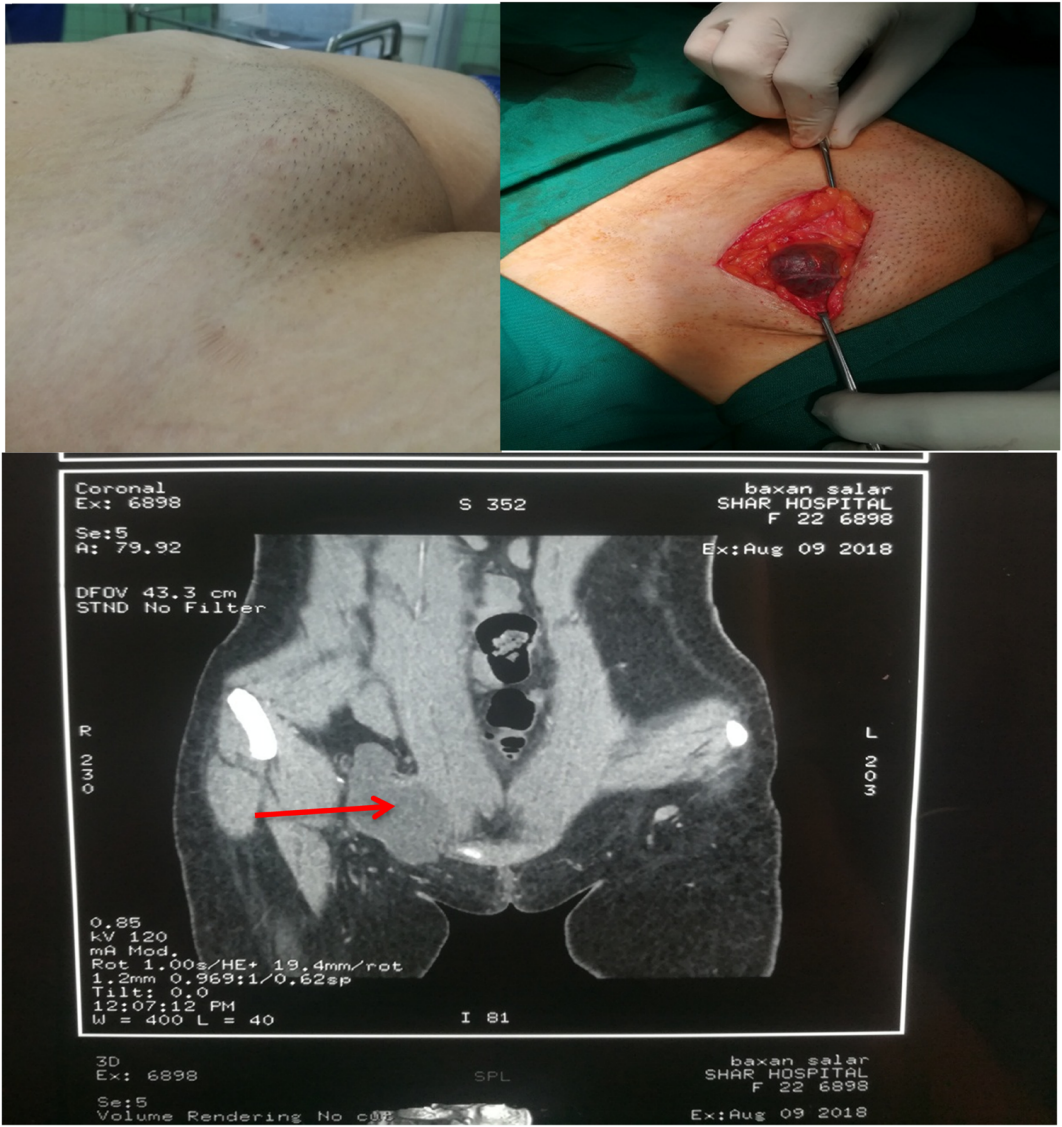
Coronal CT pelvis with IV contrast shows well well-defined lobulated cystic lesion in the right inguinal region characterized by a thin wall, no enhancing septa or solid component that was reported in patient number 2.

A 24-year-old female presented with right groin swelling for about 4 months, progressively increasing in size, without fever, rigour, or history of other masses elsewhere in the body. On examination, there was a well-defined, smooth surface, a mobile mass in the right inguinal area, negative cough impulse without inguinal lymphadenopathy, and the contralateral groin was normal. All laboratory investigations were normal. CT with IV contrast showed a well-defined thin wall lobulated cystic lesion in the right inguinal region without enhancement. Cystectomy was performed, and the final diagnosis was confirmed by histopathology examination (HPE) (Figure 2).

### 3.4 Patient number 3 with pancreatic hydatid cyst

**Figure 3:**
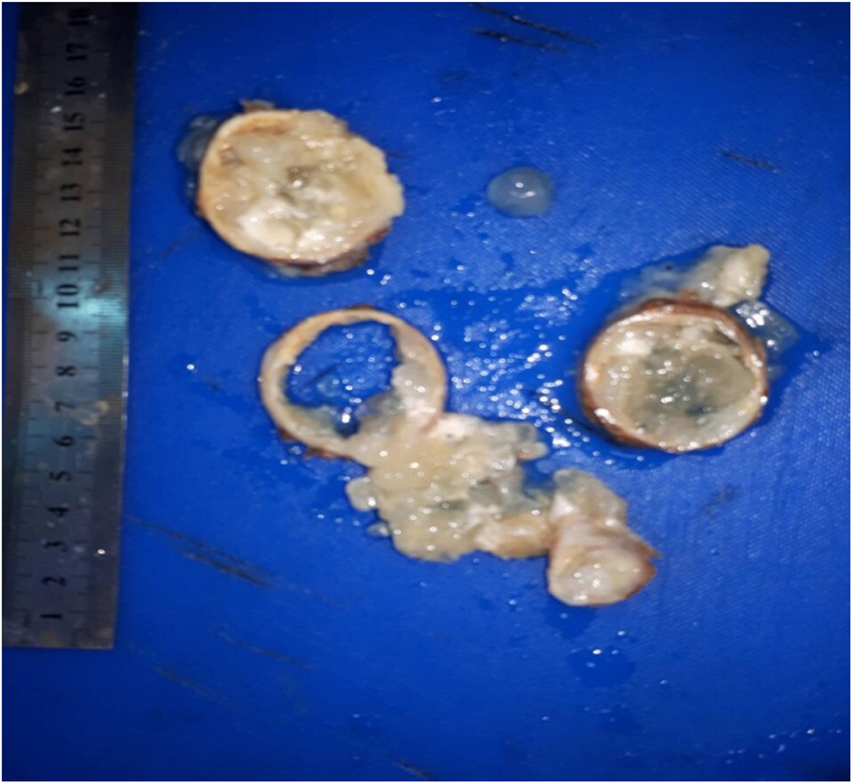
A pancreatic hydatid cyst was reported in patient number 3.

A 21-year-old female presented with intermittent upper abdominal pain for 2 months, associated with weight loss and nausea, but no vomiting or change in bowel habits. General/abdominal examinations were unremarkable, except for a thin wall with a mildly tender upper abdomen. All laboratory investigations were normal. U/S showed a complicated cyst above the pancreas gland. CT scan showed a cystic lesion at the pancreatic tail in favour of a hydatid cyst. The distal pancreatectomy was done, and the diagnosis was confirmed by HPE (Figure 3).

### 3.5 Patient number 4 with a hydatid cyst in the subcutaneous tissues of the anterior abdominal wall

**Figure 4:**
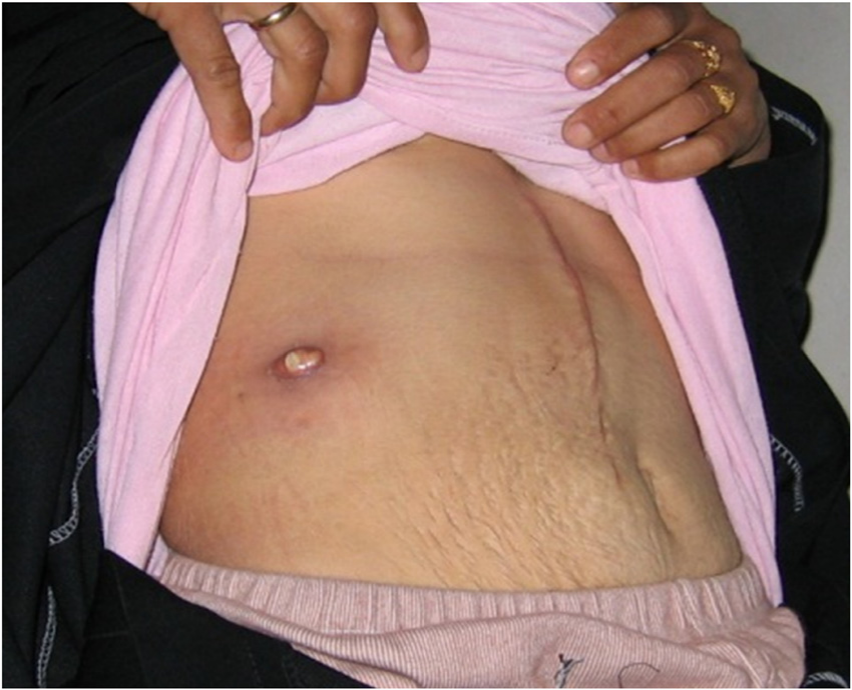
A cystic mass at the right upper abdomen associated with redness, pus discharge, and cellulitis that reported in patient number 4.

A 55-year-old female presented to the emergency department with a painful mass in the right upper abdomen. She lumped for the last 2 years at that point before admission with no fever or rigour. On examination, there was a red lump and pus, as well as surrounding cellulitis. All other abdominal studies were normal apart from the scar of upper midline laparotomy. All laboratory investigations showed normal, apart from leucocytosis. The mass was managed as an infected sebaceous cyst; the cyst was excised completely through an elliptical incision, and later, HPE revealed a hydatid cyst (Figure 4).

### 3.6 Patient number 5 with hydatid cyst of left tibial bone

**Figure 5:**
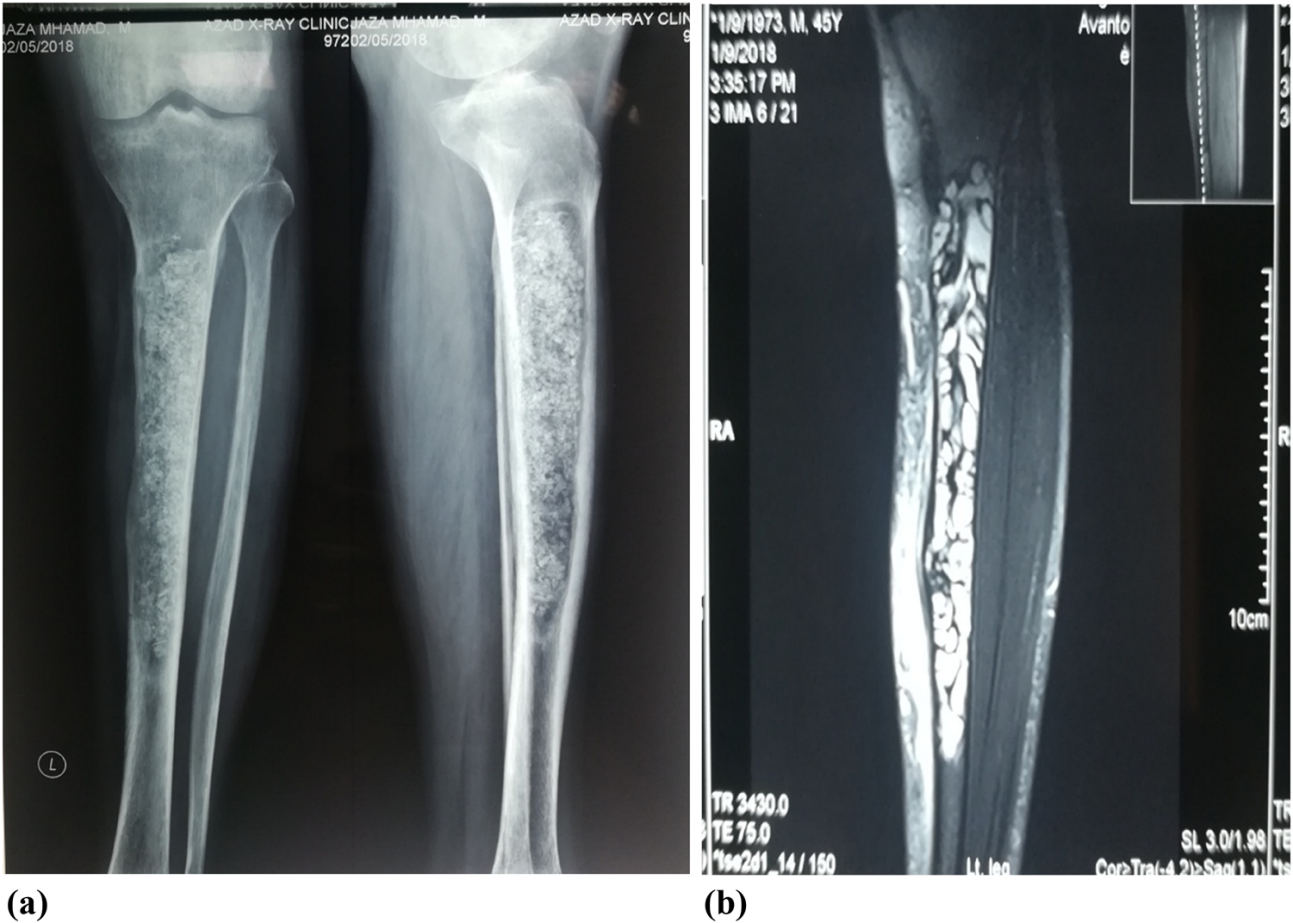
(a) A radiogram of tibial bone shows intramedullary, well-defined, diaphyseal, mixed lucent with multiple opaque shadows, mild bone expansion, no periosteal reaction, or cortical breach. (b) Coronal T2 fat suppression MRI sequence shows multiloculated cystic intramedullary diaphyseal lesion, no periosteal reaction or soft tissue component, no marrow oedema, and no fluid level. The photos are taken from patient number 5.

A 45-year-old man presented with pain in the left leg for 1 year, which was mild, aggravated by walking, and relieved by rest and pain medications. On examination of both lower limbs, the skin was normal in colour, with normal hair distribution, no dilated vein, no venous guttering, and no ulcer, while peripheral pulses dorsal pedis, anterior popliteal, popliteal, and femoral were detectable. The Doppler study ankle pressure index was normal. X-ray showed an intramedullary, well-defined, diaphyseal, mixed lucent lesion with multiple opaque shadows and mild bone expansion in the left tibia. MRI with contrast showed a multiloculated cystic intramedullary diaphyseal lesion, no periosteal reaction or soft tissue component, no marrow oedema, and no fluid level. Surgery was planned for the curation of the cyst with bone grafting, and the diagnosis of a hydatid cyst was confirmed by HPE (Figure 5).

### 3.7 Patient number 6 with a hydatid cyst in the thigh

**Figure 6:**
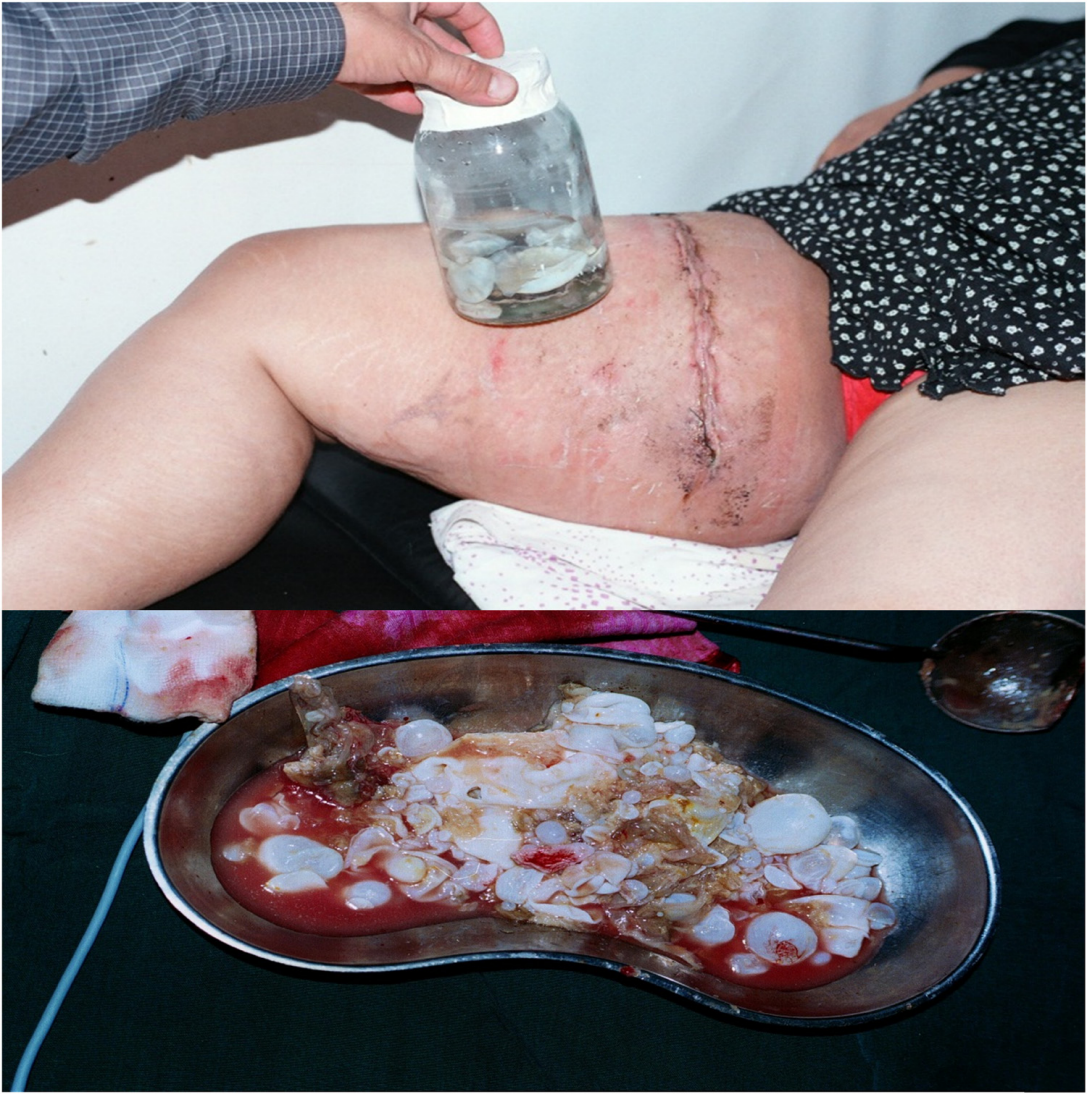
A hydatid cyst in the anterior aspect of the right thigh was reported from patient number 6.

A 39-year-old female presented with a lump in the inner aspect of the right thigh for one year, which increased in size gradually, with no history of any other lump elsewhere in the body. On examination, there was a well-defined cystic mass on the right upper thigh, not tender, with a smooth surface; the skin over it was pinchable and not trans-illuminable (Figure 6). Examination of regional lymph nodes was normal. All laboratory investigations were normal. However, U/S showed a cystic lesion with the possibility of a hydatid cyst, and the serological tests were positive for a hydatid cyst. The operation completely excised the cyst.

### 3.8 Patient number 7 with neck hydatid cyst

**Figure 7:**
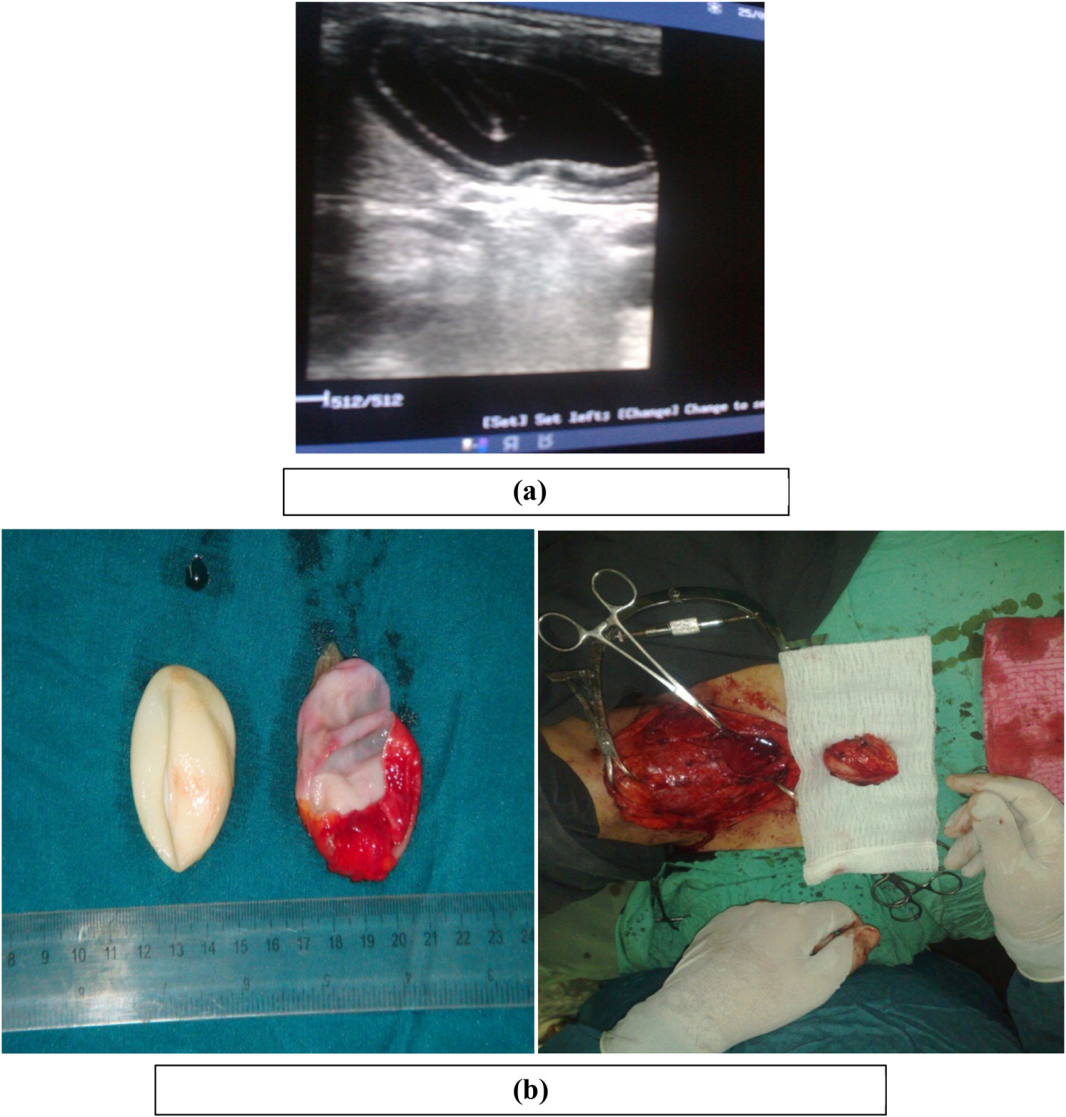
(a) Ultrasound showed a cystic lesion in the anterior neck. (b) Intraoperative picture showing hydatid cyst in the anterior aspect of the neck. The photos are taken from patient number 7.

A 31-year-old female presented with swelling in the anterior neck for 2 months but did not increase in size, had no associated symptoms (dyspnoea, hoarseness, pulse, and sweating), no change in body weight, and no tremor. On examination, there was a firm, well-defined mass at the anterior right side of the neck, smooth surface, non-tender, did not move with swallowing, and protrusion of the tongue. The regional examination of lymph nodes and thyroid gland was normal. All laboratory investigations, including thyroid function, were normal. U/S of the neck showed a cystic lesion at the anterior neck with normal cervical lymphoid and thyroid gland. A complete cyst excision was done, and HPE confirmed the diagnosis (Figure 7).

### 3.9 Patient number 8 with a hydatid cyst in the left suprarenal gland

**Figure 8:**
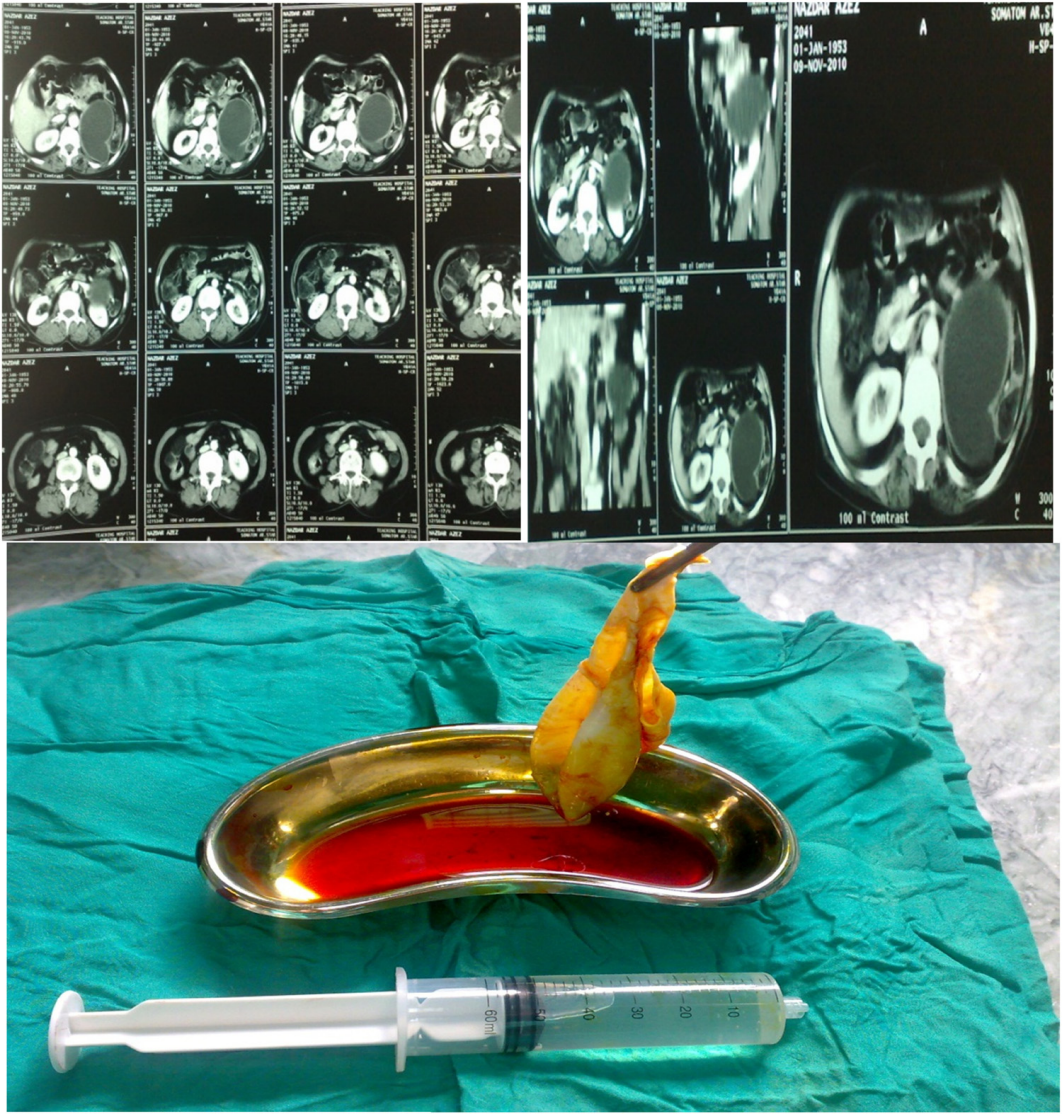
A hydatid cyst of the left suprarenal gland was reported from patient number 8.

A 60-year-old female presented with left loin pain for 2 weeks, radiating to the suprapubic region, associated with no urgency, frequency, or difficulty in urination. The abdominal examination was normal. Urinalysis and all other laboratory investigations were normal. U/S showed a thick-walled cyst at the left kidney’s upper pole, and the spleen’s posterior side measured about 104 mm × 83 mm × 75 mm. CT scan with contrast showed a large left adrenal cystic lesion. A complete excision of the cyst was done (Figure 8).

### 3.10 Patient number 9 with a hydatid cyst in the spleen

**Figure 9:**
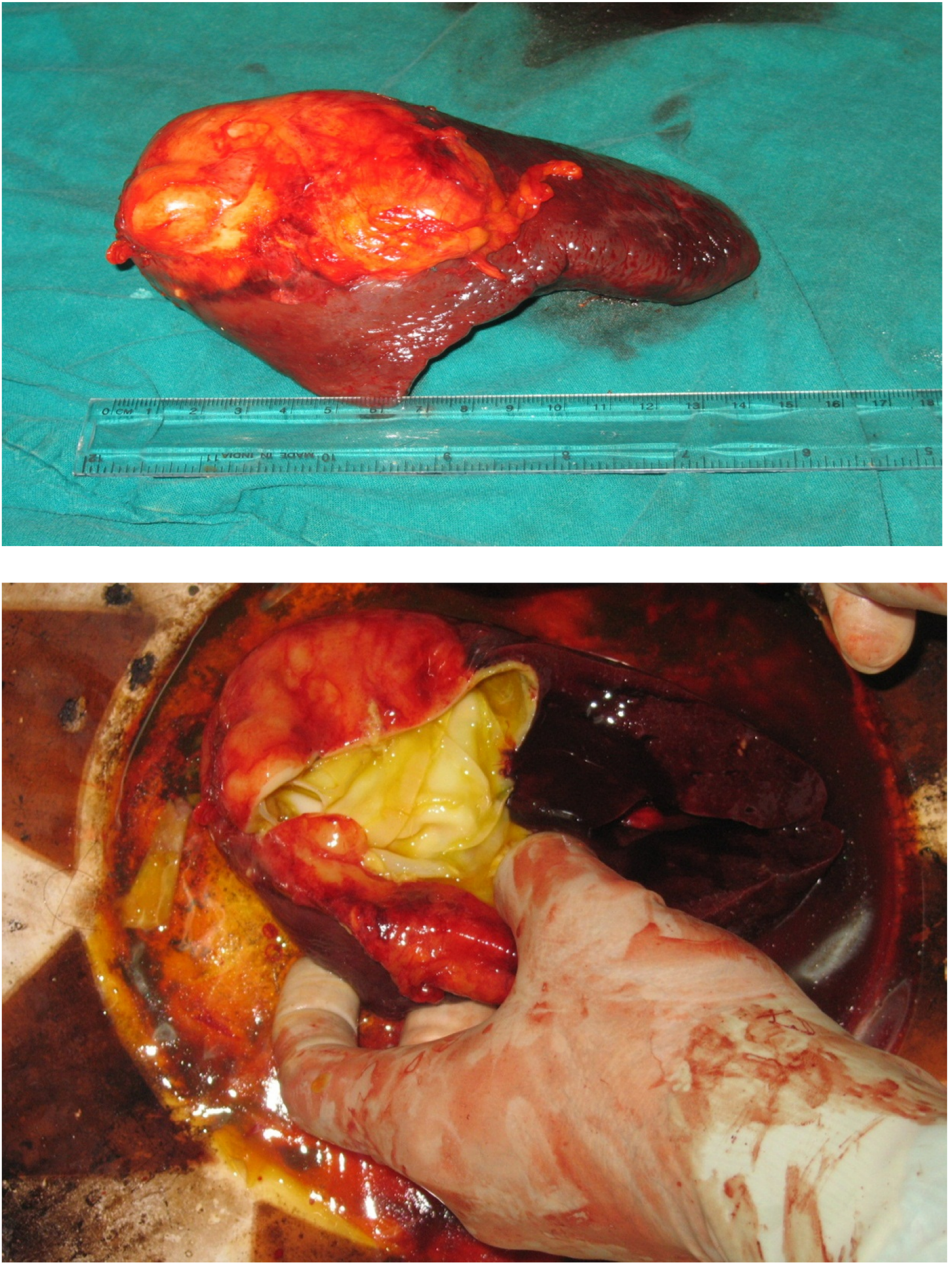
A hydatid cyst in the spleen was reported from patient number 9.

A 35-year-old female presented with mild pain in the left upper quadrant, radiating to the periumbilical region, associated with loss of appetite, but no nausea or vomiting, no change in bowel habits, and no weight loss. General/abdominal examination and all laboratory investigations were normal. However, U/S showed a cystic lesion inside the spleen. CT scan confirmed the diagnosis of a hydatid cyst. Therefore, splenectomy was performed and approved by HPE (Figure 9).

### 3.11 Patient number 10 with a hydatid cyst in the right breast

**Figure 10:**
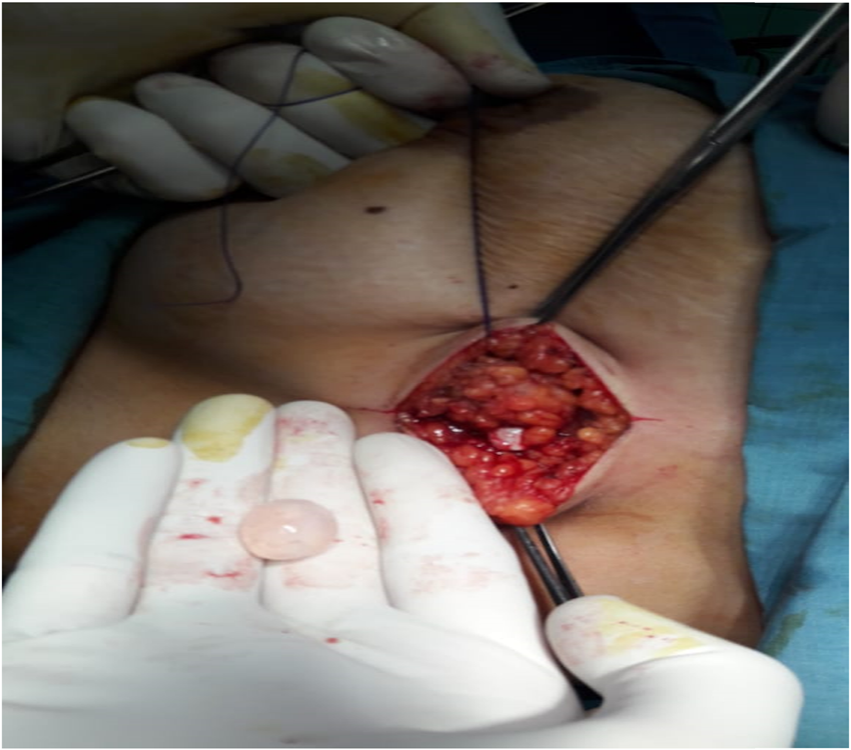
A hydatid cyst in the right breast was reported from patient number 10.

A 33-year-old female presented with a right breast mass for about 1 month, associated with discomfort and mild pain. On examination, there was a round, firm, non-tender lump at the lower inner quadrant of the right breast. No palpable axillary lymph node. U/S showed a cystic lesion containing clear fluid without axillary lymphadenopathy. The patient refused any further assessment, and a complete cyst excision was done. The final diagnosis was confirmed by HPE (Figure 10).

### 3.12 Patient number 11 with left gluteal hydatid cyst

A 38-year-old male from a rural area presented with constant pain in the left gluteal region; not radiating pain increased with time, and the pain was more during sitting and was relieved by standing. On examination, there was a tender, round, smooth surface, well-defined margin, and immobile mass at the left gluteal region without inguinal lymphadenopathy. MRI showed a cystic lesion suspicious for a hydatid cyst. ELISA was negative. HPE confirmed the final diagnosis of a hydatid cyst after complete cyst excision.

### 3.13 Patient number 12 with mesenteric hydatid cyst

A 16-year-old female presented with mild cramping abdominal pain for 7 months, associated with nausea and vomiting. General/abdominal examinations were unremarkable, with normal laboratory investigations. U/S showed an intra-abdominal cystic lesion, and a CT scan showed a cystic lesion (hydatid cyst of mesentery). The operation was done (end cystectomy), and HPE confirmed the diagnosis. During the follow-up, she was well and free from a hydatid cyst.

### 3.14 Patient number 13 with scalp hydatid cyst

A 25-year male presented with a subcutaneous mass in the scalp with no local inflammatory signs. The radiological examination was consistent with a subcutaneous cyst. Complete surgical resection of the mass was performed. Histopathological examination demonstrated hydatid cyst, and HPE confirmed the diagnosis. During the follow-up, he was well and free from a hydatid cyst.

### 3.15 Outcomes and follow-up

Within 24 years of follow-up, 12 patients were found to be cured of the disease. Only one patient developed a recurrence (tibial hydatid cyst). This patient had pain at the operation site for about 2 years associated with claudication, and investigations revealed a recurrent hydatid cyst in the tibia that was treated with re-excision.

## 4 Discussion

Although hydatid cysts are not so common in Sulaimaniyah city compared to the middle and south of Iraq, we should regard it as a differential diagnosis of any mass anywhere in the body when a mass is detected in a patient, according to the region where the individual lives and the endemicity of the disease, a hydatid cyst should be considered. Although the most common sites reported for hydatid cyst disease are the liver and the lungs, other body organs can be frequently involved.

The clinical manifestations of hydatid cysts in most parts of the body are mainly nonspecific to be diagnosed correctly based on the signs and symptoms [17,18]. In all of the previous reports from Iraq and more worldwide, serologic tests have many false-negative results. However, imaging modalities such as ultrasonography, CT scan, and MRI have been the proper methods, especially the MRI, for the preoperative diagnosis of the hydatid cyst in most unusual locations [19,20].

In this study, most patients were females, which is similar to another study in southern Iraq that enrolled 50 patients (31 females and 19 males) and organs affections in their studied patients were lungs (28.3%), liver (51.7%), spleen (6.7%), urinary bladder, kidneys, and ovaries (3.3% each), with intestine and pancreas (1.7% each). The majority (82%) had a single organ involved, while 16% had the disease in two organs, and one patient (2%) had three affected organs. Additionally, 28% of their patients aged 41–50 years and 74% were rural residents [21].

The general global reported prevalence of splenic involvement by hydatid disease varies from 0.9 to 8% [22], of which most are asymptomatic, and a minimal number of patients show nonspecific left upper quadrant pain. Splenectomy is the method of choice and is considered the gold standard for splenic hydatid cysts [23]. In this study, only one splenic hydatid cyst was found, in which the patient presented with pain in the left upper quadrant and underwent splenectomy with a good prognosis. In a study, the primary splenic hydatid cyst was found to be rare (<2%) [24], and it was reported to affect the pancreas in 0.25% of patients with hydatid disease [25].

Furthermore, this study found one pancreatic hydatid cyst involving the tail, and a distal pancreatectomy was done to remove the cyst. Generally, the pancreatic hydatid cyst is very rare, and the head of the pancreas is the most frequent location (57%) to be affected by the hydatid cyst, followed by the body (24%) and then the tail (19%) [26]. Symptoms depend on the size and site of the cyst. Cysts in the body and tail are best treated by resection methods, whereas for those in the head region, a cystectomy with simple drainage is a simple, quick, and effective solution [27]. The spread to the pancreas can be from the blood, the most common, biliary tract, intestinal lymphatics, direct passage via the pancreatic veins, and through the retroperitoneum. It is a solitary cystic lesion in around 90% of the cases. The goal is to diagnose the condition as it is scarce and similar to other pancreatic lesions on imaging. Surgery remains the preferred therapy modality [28].

Among all cases of hydatid cyst disease, 0.03–1.1% are reported to be cardiac, according to the WHO [29]. In our case series study, only one case was affected by a pericardium cyst (0.07% of the total cases). Regarding osseous hydatid cysts, the incidence is estimated to be 0.5–4%. They mostly affect a single bone, with 40–50% in the vertebral column, 25–30% in the large bones, and 15–20% in the pelvic bones. It may occur in the cranium, sternum, scapula, and phalanges, but to a much lesser extent. The most common symptom of bone hydatid cysts is pain (59%), and the main complication of surgical intervention was an infection [30]. Usually, bone disease requires long-term follow-up for recurrence, which topped at 17% in a median time of 2 years [31]. Individuals 21–30 years of age are more likely to harbour the disease than other age groups [32]. In this study, only one male patient, aged 45 years, had a left tibial hydatid cyst with recurrent disease after the first operation.

Intra-abdominal hydatid cysts, including peritoneal, omental, and mesenteric locations, are unusual. Only 49 cases were reported from 1997 to 2017, 24 from India, 11 from Turkey, and 5 from Iran [33]. In this study, two patients had mesenteric hydatid cysts; their main complaint was abdominal pain. The confirmatory test of choice was HPE. Another study points out that peritoneal hydatid cysts count for approximately 13%, and CT is the investigation of choice for diagnosis [24].

The adrenal glands are extremely rare to be involved in this condition; instead, they may get affected as part of systemic disease. The patient is usually asymptomatic or may have symptoms related to the size of the cyst [34]. Breast hydatid cysts are rare and account for 0.27% of the cases [35]. It can be primary or part of systemic disease and usually presents as a slowly enlarging painless breast mass that may mimic other causes of a breast mass. It should be included in the differential diagnosis in endemic areas. Breast hydatid cysts can be diagnosed preoperatively with serological and radiological tests combined with fine-needle aspiration. Treatment is best with complete cyst excision, but the recurrence rate might be as high as 10%, which can be lowered with post-operative Albendazole [36,37].

## 5 Conclusions

There is a common statement in Iraq saying that whenever you meet a mass anywhere in the body, if you say hydatid cyst, you are probably 50% right. Thus, the hydatid cyst can be present in any body part, and no site is immune. These unusual locations often produce nonspecific symptoms; consequently, the hydatid cyst should be considered in the differential diagnosis of all body cysts, especially in endemic countries such as Iraq.
